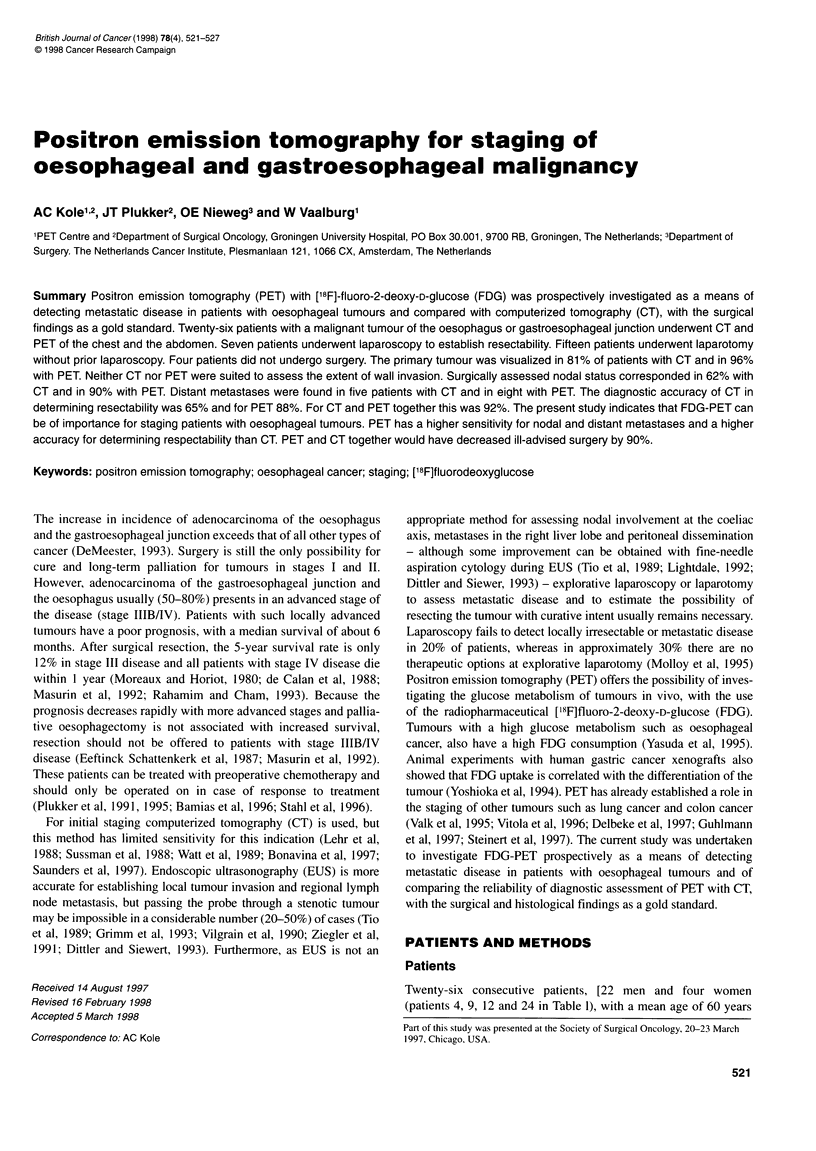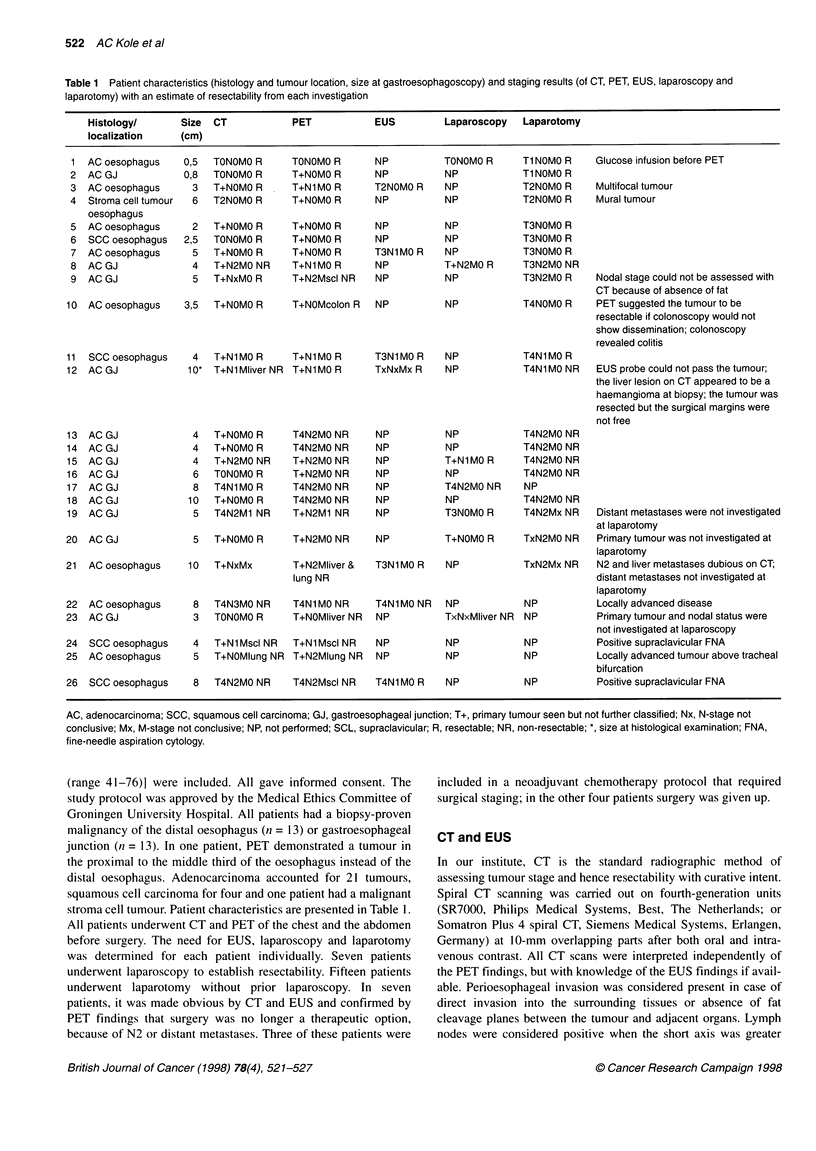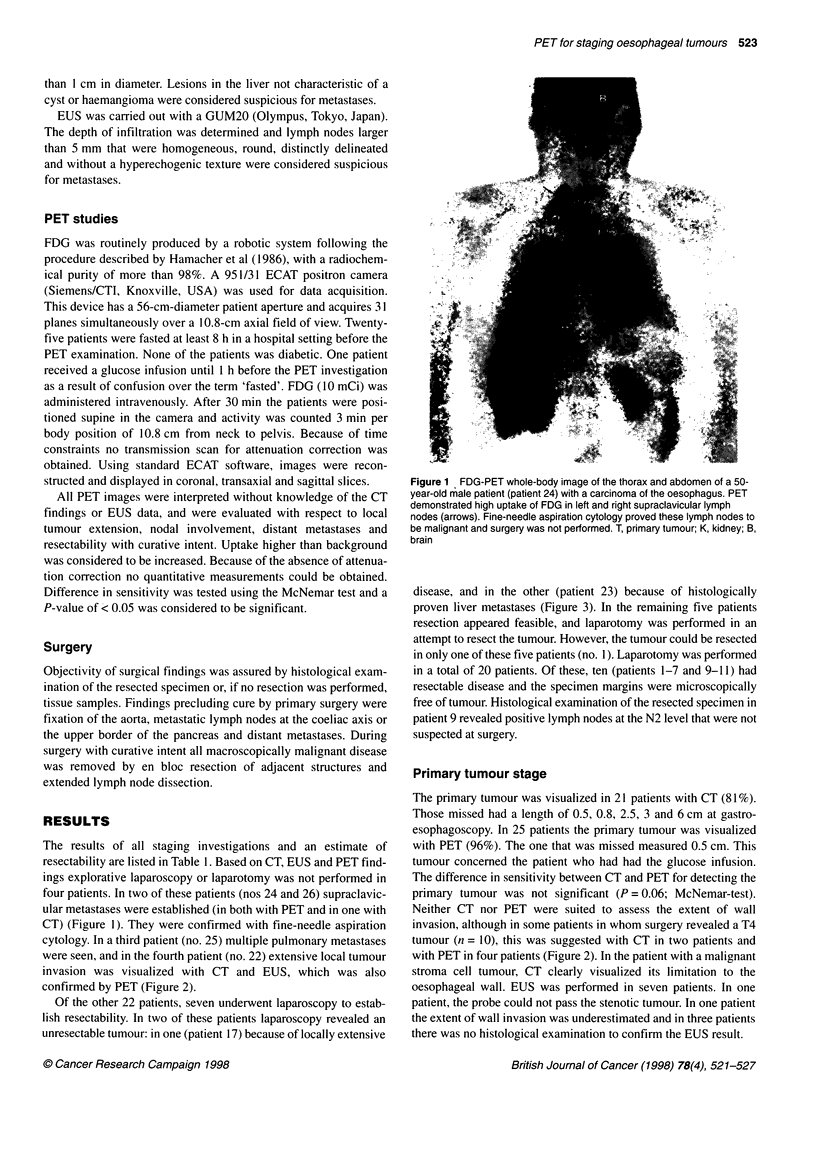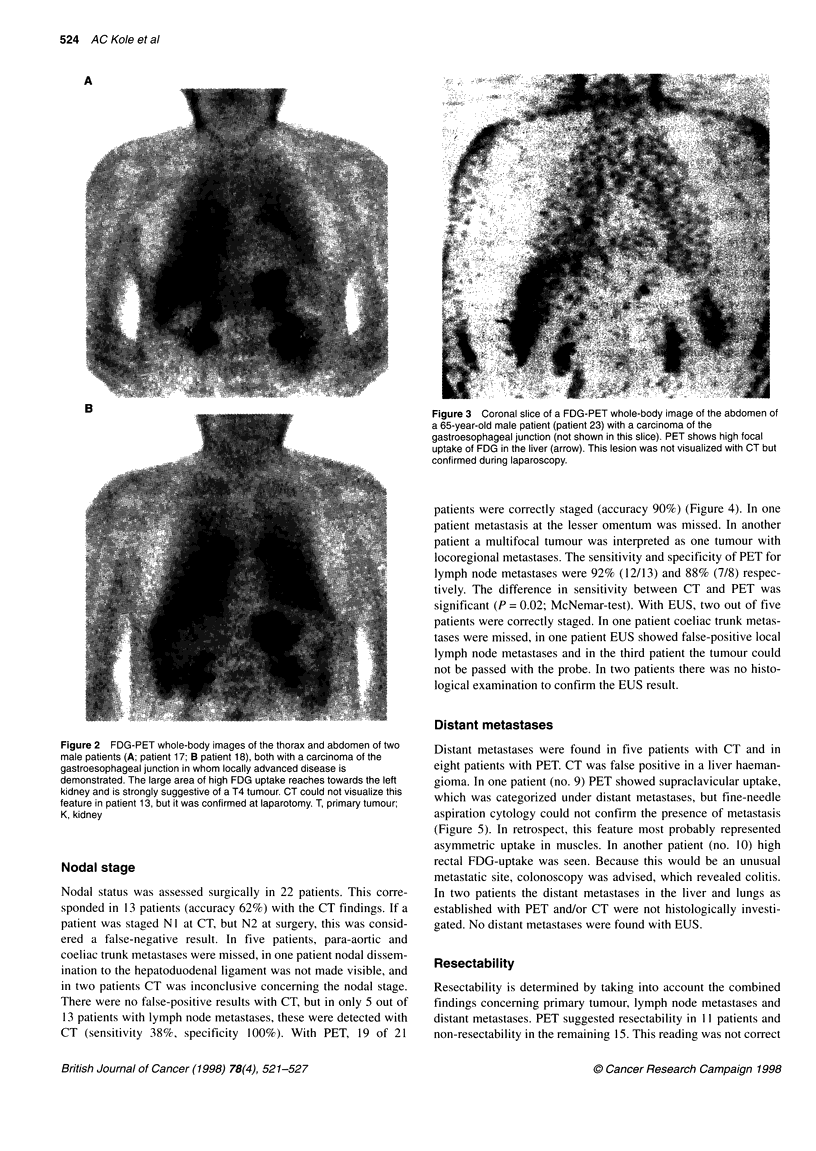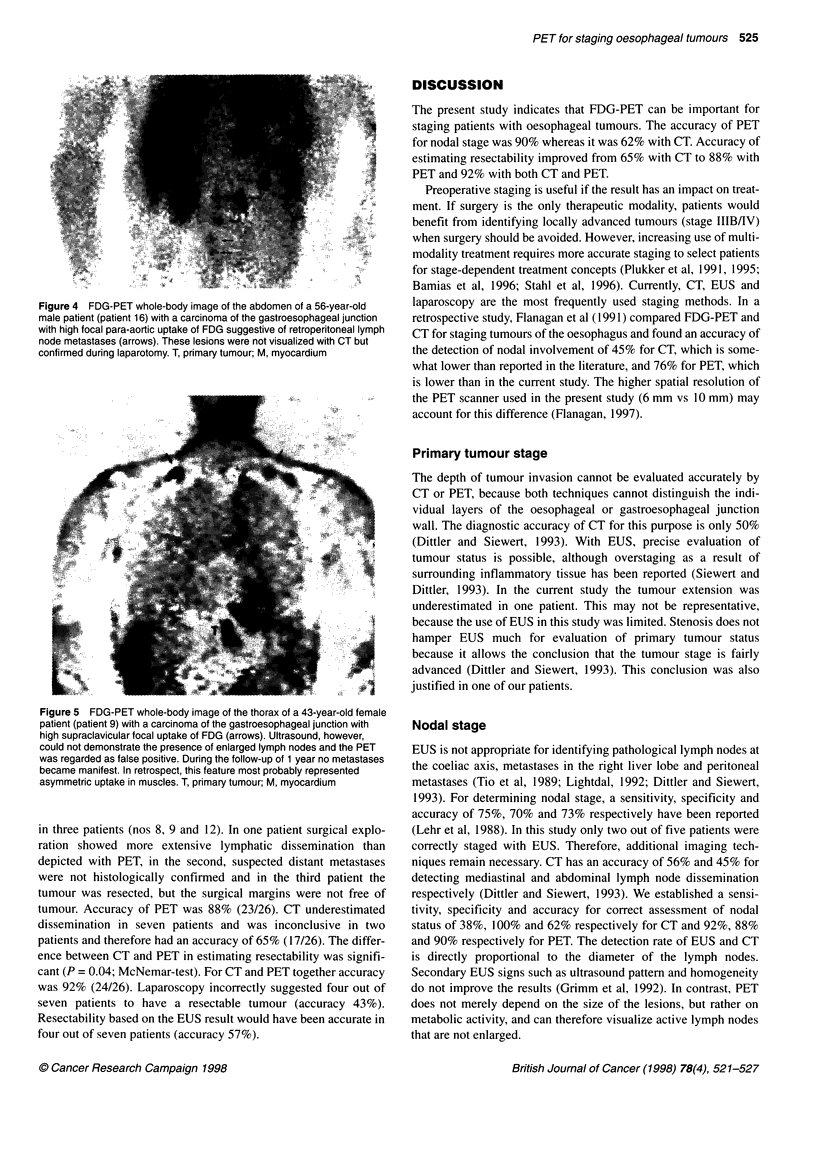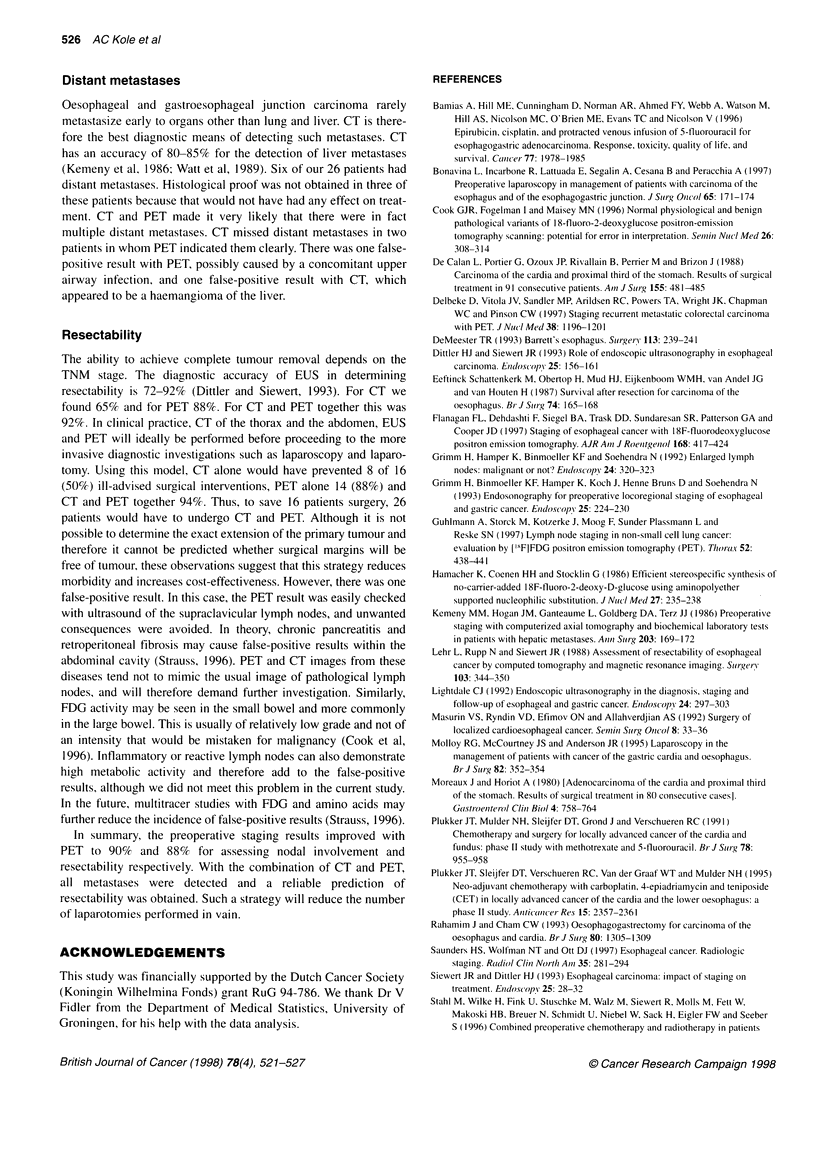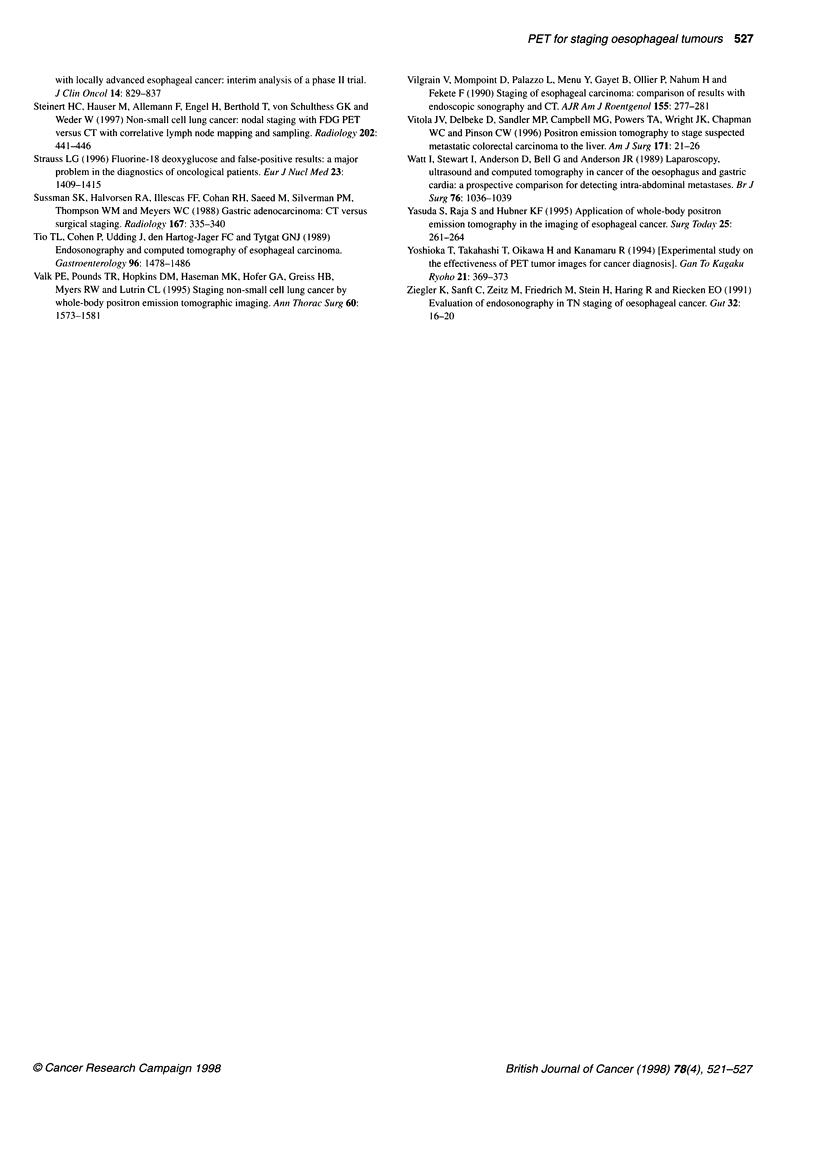# Positron emission tomography for staging of oesophageal and gastroesophageal malignancy.

**DOI:** 10.1038/bjc.1998.526

**Published:** 1998-08

**Authors:** A. C. Kole, J. T. Plukker, O. E. Nieweg, W. Vaalburg

**Affiliations:** PET Centre and Department of Surgical Oncology, Groningen University Hospital, The Netherlands.

## Abstract

**Images:**


British Journal of Cancer (1998) 78(4), 521–527
© 1998 Cancer Research Campaign

### Positron emission tomography for staging of oesophageal and gastroesophageal malignancy

**AC Kole[1,2], JT Plukker[2], OE Nieweg[3] and W Vaalburg[1]**

[1]PET Centre and [2]Department of Surgical Oncology, Groningen University Hospital, PO Box 30.001, 9700 RB, Groningen, The Netherlands; [3]Department of Surgery. The Netherlands Cancer Institute, Plesmanlaan 121, 1066 CX, Amsterdam, The Netherlands

**Summary** Positron emission tomography (PET) with [$^{18}$F]-fluoro-2-deoxy-D-glucose (FDG) was prospectively investigated as a means of detecting metastatic disease in patients with oesophageal tumours and compared with computerized tomography (CT), with the surgical findings as a gold standard. Twenty-six patients with a malignant tumour of the oesophagus or gastroesophageal junction underwent CT and PET of the chest and the abdomen. Seven patients underwent laparoscopy to establish resectability. Fifteen patients underwent laparotomy without prior laparoscopy. Four patients did not undergo surgery. The primary tumour was visualized in 81% of patients with CT and in 96% with PET. Neither CT nor PET were suited to assess the extent of wall invasion. Surgically assessed nodal status corresponded in 62% with CT and in 90% with PET. Distant metastases were found in five patients with CT and in eight with PET. The diagnostic accuracy of CT in determining resectability was 65% and for PET 88%. For CT and PET together this was 92%. The present study indicates that FDG-PET can be of importance for staging patients with oesophageal tumours. PET has a higher sensitivity for nodal and distant metastases and a higher accuracy for determining respectability than CT. PET and CT together would have decreased ill-advised surgery by 90%.

**Keywords:** positron emission tomography; oesophageal cancer; staging; [$^{18}$F]fluorodeoxyglucose

The increase in incidence of adenocarcinoma of the oesophagus and the gastroesophageal junction exceeds that of all other types of cancer (DeMeester, 1993). Surgery is still the only possibility for cure and long-term palliation for tumours in stages I and II. However, adenocarcinoma of the gastroesophageal junction and the oesophagus usually (50–80%) presents in an advanced stage of the disease (stage IIIB/IV). Patients with such locally advanced tumours have a poor prognosis, with a median survival of about 6 months. After surgical resection, the 5-year survival rate is only 12% in stage III disease and all patients with stage IV disease die within 1 year (Moreaux and Horiot, 1980; de Calan et al, 1988; Masurin et al, 1992; Rahamim and Cham, 1993). Because the prognosis decreases rapidly with more advanced stages and palliative oesophagectomy is not associated with increased survival, resection should not be offered to patients with stage IIIB/IV disease (Eeftinck Schattenkerk et al, 1987; Masurin et al, 1992). These patients can be treated with preoperative chemotherapy and should only be operated on in case of response to treatment (Plukker et al, 1991, 1995; Bamias et al, 1996; Stahl et al, 1996).

For initial staging computerized tomography (CT) is used, but this method has limited sensitivity for this indication (Lehr et al, 1988; Sussman et al, 1988; Watt et al, 1989; Bonavina et al, 1997; Saunders et al, 1997). Endoscopic ultrasonography (EUS) is more accurate for establishing local tumour invasion and regional lymph node metastasis, but passing the probe through a stenotic tumour may be impossible in a considerable number (20–50%) of cases (Tio et al, 1989; Grimm et al, 1993; Vilgrain et al, 1990; Ziegler et al, 1991; Dittler and Siewert, 1993). Furthermore, as EUS is not an appropriate method for assessing nodal involvement at the coeliac axis, metastases in the right liver lobe and peritoneal dissemination – although some improvement can be obtained with fine-needle aspiration cytology during EUS (Tio et al, 1989; Lightdale, 1992; Dittler and Siewer, 1993) – explorative laparoscopy or laparotomy to assess metastatic disease and to estimate the possibility of resecting the tumour with curative intent usually remains necessary. Laparoscopy fails to detect locally irresectable or metastatic disease in 20% of patients, whereas in approximately 30% there are no therapeutic options at explorative laparotomy (Molloy et al, 1995) Positron emission tomography (PET) offers the possibility of investigating the glucose metabolism of tumours in vivo, with the use of the radiopharmaceutical [$^{18}$F]fluoro-2-deoxy-D-glucose (FDG). Tumours with a high glucose metabolism such as oesophageal cancer, also have a high FDG consumption (Yasuda et al, 1995). Animal experiments with human gastric cancer xenografts also showed that FDG uptake is correlated with the differentiation of the tumour (Yoshioka et al, 1994). PET has already established a role in the staging of other tumours such as lung cancer and colon cancer (Valk et al, 1995; Vitola et al, 1996; Delbeke et al, 1997; Guhlmann et al, 1997; Steinert et al, 1997). The current study was undertaken to investigate FDG-PET prospectively as a means of detecting metastatic disease in patients with oesophageal tumours and of comparing the reliability of diagnostic assessment of PET with CT, with the surgical and histological findings as a gold standard.

### PATIENTS AND METHODS

### Patients

Twenty-six consecutive patients, [22 men and four women (patients 4, 9, 12 and 24 in Table 1), with a mean age of 60 years

*Received 14 August 1997*
*Revised 16 February 1998*
*Accepted 5 March 1998*

*Correspondence to:* AC Kole

Part of this study was presented at the Society of Surgical Oncology, 20–23 March 1997, Chicago, USA.

**Table 1** Patient characteristics (histology and tumour location, size at gastroesophagoscopy) and staging results (of CT, PET, EUS, laparoscopy and laparotomy) with an estimate of resectability from each investigation

| | Histology/ localization | Size (cm) | CT | PET | EUS | Laparoscopy | Laparotomy | |
|---|---|---|---|---|---|---|---|---|
| 1 | AC oesophagus | 0,5 | T0N0M0 R | T0N0M0 R | NP | T0N0M0 R | T1N0M0 R | Glucose infusion before PET |
| 2 | AC GJ | 0,8 | T0N0M0 R | T+N0M0 R | NP | NP | T1N0M0 R | |
| 3 | AC oesophagus | 3 | T+N0M0 R | T+N1M0 R | T2N0M0 R | NP | T2N0M0 R | Multifocal tumour |
| 4 | Stroma cell tumour oesophagus | 6 | T2N0M0 R | T+N0M0 R | NP | NP | T2N0M0 R | Mural tumour |
| 5 | AC oesophagus | 2 | T+N0M0 R | T+N0M0 R | NP | NP | T3N0M0 R | |
| 6 | SCC oesophagus | 2,5 | T0N0M0 R | T+N0M0 R | NP | NP | T3N0M0 R | |
| 7 | AC oesophagus | 5 | T+N0M0 R | T+N0M0 R | T3N1M0 R | NP | T3N0M0 R | |
| 8 | AC GJ | 4 | T+N2M0 NR | T+N1M0 R | NP | T+N2M0 R | T3N2M0 NR | |
| 9 | AC GJ | 5 | T+NxM0 R | T+N2Mscl NR | NP | NP | T3N2M0 R | Nodal stage could not be assessed with CT because of absence of fat |
| 10 | AC oesophagus | 3,5 | T+N0M0 R | T+N0Mcolon R | NP | NP | T4N0M0 R | PET suggested the tumour to be resectable if colonoscopy would not show dissemination; colonoscopy revealed colitis |
| 11 | SCC oesophagus | 4 | T+N1M0 R | T+N1M0 R | T3N1M0 R | NP | T4N1M0 R | |
| 12 | AC GJ | 10* | T+N1Mliver NR | T+N1M0 R | TxNxMx R | NP | T4N1M0 NR | EUS probe could not pass the tumour; the liver lesion on CT appeared to be a haemangioma at biopsy; the tumour was resected but the surgical margins were not free |
| 13 | AC GJ | 4 | T+N0M0 R | T4N2M0 NR | NP | NP | T4N2M0 NR | |
| 14 | AC GJ | 4 | T+N0M0 R | T4N2M0 NR | NP | NP | T4N2M0 NR | |
| 15 | AC GJ | 4 | T+N2M0 NR | T+N2M0 NR | NP | T+N1M0 R | T4N2M0 NR | |
| 16 | AC GJ | 6 | T0N0M0 R | T+N2M0 NR | NP | NP | T4N2M0 NR | |
| 17 | AC GJ | 8 | T4N1M0 R | T4N2M0 NR | NP | T4N2M0 NR | NP | |
| 18 | AC GJ | 10 | T+N0M0 R | T4N2M0 NR | NP | NP | T4N2M0 NR | |
| 19 | AC GJ | 5 | T4N2M1 NR | T+N2M1 NR | NP | T3N0M0 R | T4N2Mx NR | Distant metastases were not investigated at laparotomy |
| 20 | AC GJ | 5 | T+N0M0 R | T+N2M0 NR | NP | T+N0M0 R | TxN2M0 NR | Primary tumour was not investigated at laparotomy |
| 21 | AC oesophagus | 10 | T+NxMx | T+N2Mliver & lung NR | T3N1M0 R | NP | TxN2Mx NR | N2 and liver metastases dubious on CT; distant metastases not investigated at laparotomy |
| 22 | AC oesophagus | 8 | T4N3M0 NR | T4N1M0 NR | T4N1M0 NR | NP | NP | Locally advanced disease |
| 23 | AC GJ | 3 | T0N0M0 R | T+N0Mliver NR | NP | TxNxMliver NR | NP | Primary tumour and nodal status were not investigated at laparoscopy |
| 24 | SCC oesophagus | 4 | T+N1Mscl NR | T+N1Mscl NR | NP | NP | NP | Positive supraclavicular FNA |
| 25 | AC oesophagus | 5 | T+N0Mlung NR | T+N2Mlung NR | NP | NP | NP | Locally advanced tumour above tracheal bifurcation |
| 26 | SCC oesophagus | 8 | T4N2M0 NR | T4N2Mscl NR | T4N1M0 R | NP | NP | Positive supraclavicular FNA |

AC, adenocarcinoma; SCC, squamous cell carcinoma; GJ, gastroesophageal junction; T+, primary tumour seen but not further classified; Nx, N-stage not conclusive; Mx, M-stage not conclusive; NP, not performed; SCL, supraclavicular; R, resectable; NR, non-resectable; *, size at histological examination; FNA, fine-needle aspiration cytology.

(range 41–76)] were included. All gave informed consent. The study protocol was approved by the Medical Ethics Committee of Groningen University Hospital. All patients had a biopsy-proven malignancy of the distal oesophagus ($n$ = 13) or gastroesophageal junction ($n$ = 13). In one patient, PET demonstrated a tumour in the proximal to the middle third of the oesophagus instead of the distal oesophagus. Adenocarcinoma accounted for 21 tumours, squamous cell carcinoma for four and one patient had a malignant stroma cell tumour. Patient characteristics are presented in Table 1. All patients underwent CT and PET of the chest and the abdomen before surgery. The need for EUS, laparoscopy and laparotomy was determined for each patient individually. Seven patients underwent laparoscopy to establish resectability. Fifteen patients underwent laparotomy without prior laparoscopy. In seven patients, it was made obvious by CT and EUS and confirmed by PET findings that surgery was no longer a therapeutic option, because of N2 or distant metastases. Three of these patients were included in a neoadjuvant chemotherapy protocol that required surgical staging; in the other four patients surgery was given up.

### CT and EUS

In our institute, CT is the standard radiographic method of assessing tumour stage and hence resectability with curative intent. Spiral CT scanning was carried out on fourth-generation units (SR7000, Philips Medical Systems, Best, The Netherlands; or Somatron Plus 4 spiral CT, Siemens Medical Systems, Erlangen, Germany) at 10-mm overlapping parts after both oral and intravenous contrast. All CT scans were interpreted independently of the PET findings, but with knowledge of the EUS findings if available. Perioesophageal invasion was considered present in case of direct invasion into the surrounding tissues or absence of fat cleavage planes between the tumour and adjacent organs. Lymph nodes were considered positive when the short axis was greater

© Cancer Research Campaign 1998

than 1 cm in diameter. Lesions in the liver not characteristic of a cyst or haemangioma were considered suspicious for metastases.

EUS was carried out with a GUM20 (Olympus, Tokyo, Japan). The depth of infiltration was determined and lymph nodes larger than 5 mm that were homogeneous, round, distinctly delineated and without a hyperechogenic texture were considered suspicious for metastases.

### PET studies

FDG was routinely produced by a robotic system following the procedure described by Hamacher et al (1986), with a radiochemical purity of more than 98%. A 951/31 ECAT positron camera (Siemens/CTI, Knoxville, USA) was used for data acquisition. This device has a 56-cm-diameter patient aperture and acquires 31 planes simultaneously over a 10.8-cm axial field of view. Twenty-five patients were fasted at least 8 h in a hospital setting before the PET examination. None of the patients was diabetic. One patient received a glucose infusion until 1 h before the PET investigation as a result of confusion over the term ‘fasted’. FDG (10 mCi) was administered intravenously. After 30 min the patients were positioned supine in the camera and activity was counted 3 min per body position of 10.8 cm from neck to pelvis. Because of time constraints no transmission scan for attenuation correction was obtained. Using standard ECAT software, images were reconstructed and displayed in coronal, transaxial and sagittal slices.

All PET images were interpreted without knowledge of the CT findings or EUS data, and were evaluated with respect to local tumour extension, nodal involvement, distant metastases and resectability with curative intent. Uptake higher than background was considered to be increased. Because of the absence of attenuation correction no quantitative measurements could be obtained. Difference in sensitivity was tested using the McNemar test and a $P$-value of $< 0.05$ was considered to be significant.

### Surgery

Objectivity of surgical findings was assured by histological examination of the resected specimen or, if no resection was performed, tissue samples. Findings precluding cure by primary surgery were fixation of the aorta, metastatic lymph nodes at the coeliac axis or the upper border of the pancreas and distant metastases. During surgery with curative intent all macroscopically malignant disease was removed by en bloc resection of adjacent structures and extended lymph node dissection.

### RESULTS

The results of all staging investigations and an estimate of resectability are listed in Table 1. Based on CT, EUS and PET findings explorative laparoscopy or laparotomy was not performed in four patients. In two of these patients (nos 24 and 26) supraclavicular metastases were established (in both with PET and in one with CT) (Figure 1). They were confirmed with fine-needle aspiration cytology. In a third patient (no. 25) multiple pulmonary metastases were seen, and in the fourth patient (no. 22) extensive local tumour invasion was visualized with CT and EUS, which was also confirmed by PET (Figure 2).

Of the other 22 patients, seven underwent laparoscopy to establish resectability. In two of these patients laparoscopy revealed an unresectable tumour: in one (patient 17) because of locally extensive disease, and in the other (patient 23) because of histologically proven liver metastases (Figure 3). In the remaining five patients resection appeared feasible, and laparotomy was performed in an attempt to resect the tumour. However, the tumour could be resected in only one of these five patients (no. 1). Laparotomy was performed in a total of 20 patients. Of these, ten (patients 1–7 and 9–11) had resectable disease and the specimen margins were microscopically free of tumour. Histological examination of the resected specimen in patient 9 revealed positive lymph nodes at the N2 level that were not suspected at surgery.

**Figure 1** FDG-PET whole-body image of the thorax and abdomen of a 50-year-old male patient (patient 24) with a carcinoma of the oesophagus. PET demonstrated high uptake of FDG in left and right supraclavicular lymph nodes (arrows). Fine-needle aspiration cytology proved these lymph nodes to be malignant and surgery was not performed. T, primary tumour; K, kidney; B, brain

### Primary tumour stage

The primary tumour was visualized in 21 patients with CT (81%). Those missed had a length of 0.5, 0.8, 2.5, 3 and 6 cm at gastro-esophagoscopy. In 25 patients the primary tumour was visualized with PET (96%). The one that was missed measured 0.5 cm. This tumour concerned the patient who had had the glucose infusion. The difference in sensitivity between CT and PET for detecting the primary tumour was not significant ($P = 0.06$; McNemar-test). Neither CT nor PET were suited to assess the extent of wall invasion, although in some patients in whom surgery revealed a T4 tumour ($n = 10$), this was suggested with CT in two patients and with PET in four patients (Figure 2). In the patient with a malignant stroma cell tumour, CT clearly visualized its limitation to the oesophageal wall. EUS was performed in seven patients. In one patient, the probe could not pass the stenotic tumour. In one patient the extent of wall invasion was underestimated and in three patients there was no histological examination to confirm the EUS result.

© Cancer Research Campaign 1998

Figure 2 FDG-PET whole-body images of the thorax and abdomen of two male patients (**A**; patient 17; **B** patient 18), both with a carcinoma of the gastroesophageal junction in whom locally advanced disease is demonstrated. The large area of high FDG uptake reaches towards the left kidney and is strongly suggestive of a T4 tumour. CT could not visualize this feature in patient 13, but it was confirmed at laparotomy. T, primary tumour; K, kidney

**Figure 3** Coronal slice of a FDG-PET whole-body image of the abdomen of a 65-year-old male patient (patient 23) with a carcinoma of the gastroesophageal junction (not shown in this slice). PET shows high focal uptake of FDG in the liver (arrow). This lesion was not visualized with CT but confirmed during laparoscopy.

### Nodal stage

Nodal status was assessed surgically in 22 patients. This corresponded in 13 patients (accuracy 62%) with the CT findings. If a patient was staged N1 at CT, but N2 at surgery, this was considered a false-negative result. In five patients, para-aortic and coeliac trunk metastases were missed, in one patient nodal dissemination to the hepatoduodenal ligament was not made visible, and in two patients CT was inconclusive concerning the nodal stage. There were no false-positive results with CT, but in only 5 out of 13 patients with lymph node metastases, these were detected with CT (sensitivity 38%, specificity 100%). With PET, 19 of 21 patients were correctly staged (accuracy 90%) (Figure 4). In one patient metastasis at the lesser omentum was missed. In another patient a multifocal tumour was interpreted as one tumour with locoregional metastases. The sensitivity and specificity of PET for lymph node metastases were 92% (12/13) and 88% (7/8) respectively. The difference in sensitivity between CT and PET was significant ($P = 0.02$; McNemar-test). With EUS, two out of five patients were correctly staged. In one patient coeliac trunk metastases were missed, in one patient EUS showed false-positive local lymph node metastases and in the third patient the tumour could not be passed with the probe. In two patients there was no histological examination to confirm the EUS result.

### Distant metastases

Distant metastases were found in five patients with CT and in eight patients with PET. CT was false positive in a liver haemangioma. In one patient (no. 9) PET showed supraclavicular uptake, which was categorized under distant metastases, but fine-needle aspiration cytology could not confirm the presence of metastasis (Figure 5). In retrospect, this feature most probably represented asymmetric uptake in muscles. In another patient (no. 10) high rectal FDG-uptake was seen. Because this would be an unusual metastatic site, colonoscopy was advised, which revealed colitis. In two patients the distant metastases in the liver and lungs as established with PET and/or CT were not histologically investigated. No distant metastases were found with EUS.

### Resectability

Resectability is determined by taking into account the combined findings concerning primary tumour, lymph node metastases and distant metastases. PET suggested resectability in 11 patients and non-resectability in the remaining 15. This reading was not correct

 © Cancer Research Campaign 1998

**Figure 4** FDG-PET whole-body image of the abdomen of a 56-year-old male patient (patient 16) with a carcinoma of the gastroesophageal junction with high focal para-aortic uptake of FDG suggestive of retroperitoneal lymph node metastases (arrows). These lesions were not visualized with CT but confirmed during laparotomy. T, primary tumour; M, myocardium

**Figure 5** FDG-PET whole-body image of the thorax of a 43-year-old female patient (patient 9) with a carcinoma of the gastroesophageal junction with high supraclavicular focal uptake of FDG (arrows). Ultrasound, however, could not demonstrate the presence of enlarged lymph nodes and the PET was regarded as false positive. During the follow-up of 1 year no metastases became manifest. In retrospect, this feature most probably represented asymmetric uptake in muscles. T, primary tumour; M, myocardium in three patients (nos 8, 9 and 12). In one patient surgical exploration showed more extensive lymphatic dissemination than depicted with PET, in the second, suspected distant metastases were not histologically confirmed and in the third patient the tumour was resected, but the surgical margins were not free of tumour. Accuracy of PET was 88% (23/26). CT underestimated dissemination in seven patients and was inconclusive in two patients and therefore had an accuracy of 65% (17/26). The difference between CT and PET in estimating resectability was significant ($P = 0.04$; McNemar-test). For CT and PET together accuracy was 92% (24/26). Laparoscopy incorrectly suggested four out of seven patients to have a resectable tumour (accuracy 43%). Resectability based on the EUS result would have been accurate in four out of seven patients (accuracy 57%).

### DISCUSSION

The present study indicates that FDG-PET can be important for staging patients with oesophageal tumours. The accuracy of PET for nodal stage was 90% whereas it was 62% with CT. Accuracy of estimating resectability improved from 65% with CT to 88% with PET and 92% with both CT and PET.

Preoperative staging is useful if the result has an impact on treatment. If surgery is the only therapeutic modality, patients would benefit from identifying locally advanced tumours (stage IIIB/IV) when surgery should be avoided. However, increasing use of multimodality treatment requires more accurate staging to select patients for stage-dependent treatment concepts (Plukker et al, 1991, 1995; Bamias et al, 1996; Stahl et al, 1996). Currently, CT, EUS and laparoscopy are the most frequently used staging methods. In a retrospective study, Flanagan et al (1991) compared FDG-PET and CT for staging tumours of the oesophagus and found an accuracy of the detection of nodal involvement of 45% for CT, which is somewhat lower than reported in the literature, and 76% for PET, which is lower than in the current study. The higher spatial resolution of the PET scanner used in the present study (6 mm vs 10 mm) may account for this difference (Flanagan, 1997).

### Primary tumour stage

The depth of tumour invasion cannot be evaluated accurately by CT or PET, because both techniques cannot distinguish the individual layers of the oesophageal or gastroesophageal junction wall. The diagnostic accuracy of CT for this purpose is only 50% (Dittler and Siewert, 1993). With EUS, precise evaluation of tumour status is possible, although overstaging as a result of surrounding inflammatory tissue has been reported (Siewert and Dittler, 1993). In the current study the tumour extension was underestimated in one patient. This may not be representative, because the use of EUS in this study was limited. Stenosis does not hamper EUS much for evaluation of primary tumour status because it allows the conclusion that the tumour stage is fairly advanced (Dittler and Siewert, 1993). This conclusion was also justified in one of our patients.

### Nodal stage

EUS is not appropriate for identifying pathological lymph nodes at the coeliac axis, metastases in the right liver lobe and peritoneal metastases (Tio et al, 1989; Lightdal, 1992; Dittler and Siewert, 1993). For determining nodal stage, a sensitivity, specificity and accuracy of 75%, 70% and 73% respectively have been reported (Lehr et al, 1988). In this study only two out of five patients were correctly staged with EUS. Therefore, additional imaging techniques remain necessary. CT has an accuracy of 56% and 45% for detecting mediastinal and abdominal lymph node dissemination respectively (Dittler and Siewert, 1993). We established a sensitivity, specificity and accuracy for correct assessment of nodal status of 38%, 100% and 62% respectively for CT and 92%, 88% and 90% respectively for PET. The detection rate of EUS and CT is directly proportional to the diameter of the lymph nodes. Secondary EUS signs such as ultrasound pattern and homogeneity do not improve the results (Grimm et al, 1992). In contrast, PET does not merely depend on the size of the lesions, but rather on metabolic activity, and can therefore visualize active lymph nodes that are not enlarged.

© Cancer Research Campaign 1998

### Distant metastases

Oesophageal and gastroesophageal junction carcinoma rarely metastasize early to organs other than lung and liver. CT is therefore the best diagnostic means of detecting such metastases. CT has an accuracy of 80–85% for the detection of liver metastases (Kemeny et al, 1986; Watt et al, 1989). Six of our 26 patients had distant metastases. Histological proof was not obtained in three of these patients because that would not have had any effect on treatment. CT and PET made it very likely that there were in fact multiple distant metastases. CT missed distant metastases in two patients in whom PET indicated them clearly. There was one false-positive result with PET, possibly caused by a concomitant upper airway infection, and one false-positive result with CT, which appeared to be a haemangioma of the liver.

### Resectability

The ability to achieve complete tumour removal depends on the TNM stage. The diagnostic accuracy of EUS in determining resectability is 72–92% (Dittler and Siewert, 1993). For CT we found 65% and for PET 88%. For CT and PET together this was 92%. In clinical practice, CT of the thorax and the abdomen, EUS and PET will ideally be performed before proceeding to the more invasive diagnostic investigations such as laparoscopy and laparotomy. Using this model, CT alone would have prevented 8 of 16 (50%) ill-advised surgical interventions, PET alone 14 (88%) and CT and PET together 94%. Thus, to save 16 patients surgery, 26 patients would have to undergo CT and PET. Although it is not possible to determine the exact extension of the primary tumour and therefore it cannot be predicted whether surgical margins will be free of tumour, these observations suggest that this strategy reduces morbidity and increases cost-effectiveness. However, there was one false-positive result. In this case, the PET result was easily checked with ultrasound of the supraclavicular lymph nodes, and unwanted consequences were avoided. In theory, chronic pancreatitis and retroperitoneal fibrosis may cause false-positive results within the abdominal cavity (Strauss, 1996). PET and CT images from these diseases tend not to mimic the usual image of pathological lymph nodes, and will therefore demand further investigation. Similarly, FDG activity may be seen in the small bowel and more commonly in the large bowel. This is usually of relatively low grade and not of an intensity that would be mistaken for malignancy (Cook et al, 1996). Inflammatory or reactive lymph nodes can also demonstrate high metabolic activity and therefore add to the false-positive results, although we did not meet this problem in the current study. In the future, multitracer studies with FDG and amino acids may further reduce the incidence of false-positive results (Strauss, 1996).

In summary, the preoperative staging results improved with PET to 90% and 88% for assessing nodal involvement and resectability respectively. With the combination of CT and PET, all metastases were detected and a reliable prediction of resectability was obtained. Such a strategy will reduce the number of laparotomies performed in vain.

### ACKNOWLEDGEMENTS

This study was financially supported by the Dutch Cancer Society (Koningin Wilhelmina Fonds) grant RuG 94-786. We thank Dr V Fidler from the Department of Medical Statistics, University of Groningen, for his help with the data analysis.

### REFERENCES

Bamias A, Hill ME, Cunningham D, Norman AR, Ahmed FY, Webb A, Watson M, Hill AS, Nicolson MC, O'Brien ME, Evans TC and Nicolson V (1996) Epirubicin, cisplatin, and protracted venous infusion of 5-fluorouracil for esophagogastric adenocarcinoma. Response, toxicity, quality of life, and survival. *Cancer* **77**: 1978–1985

Bonavina L, Incarbone R, Lattuada E, Segalin A, Cesana B and Peracchia A (1997) Preoperative laparoscopy in management of patients with carcinoma of the esophagus and of the esophagogastric junction. *J Surg Oncol* **65**: 171–174

Cook GJR, Fogelman I and Maisey MN (1996) Normal physiological and benign pathological variants of 18-fluoro-2-deoxyglucose positron-emission tomography scanning: potential for error in interpretation. *Semin Nucl Med* **26**: 308–314

De Calan L, Portier G, Ozoux JP, Rivallain B, Perrier M and Brizon J (1988) Carcinoma of the cardia and proximal third of the stomach. Results of surgical treatment in 91 consecutive patients. *Am J Surg* **155**: 481–485

Delbeke D, Vitola JV, Sandler MP, Arildsen RC, Powers TA, Wright JK, Chapman WC and Pinson CW (1997) Staging recurrent metastatic colorectal carcinoma with PET. *J Nucl Med* **38**: 1196–1201

DeMeester TR (1993) Barrett's esophagus. *Surgery* **113**: 239–241

Dittler HJ and Siewert JR (1993) Role of endoscopic ultrasonography in esophageal carcinoma. *Endoscopy* **25**: 156–161

Eeftinck Schattenkerk M, Obertop H, Mud HJ, Eijkenboom WMH, van Andel JG and van Houten H (1987) Survival after resection for carcinoma of the oesophagus. *Br J Surg* **74**: 165–168

Flanagan FL, Dehdashti F, Siegel BA, Trask DD, Sundaresan SR, Patterson GA and Cooper JD (1997) Staging of esophageal cancer with 18F-fluorodeoxyglucose positron emission tomography. *AJR Am J Roentgenol* **168**: 417–424

Grimm H, Hamper K, Binmoeller KF and Soehendra N (1992) Enlarged lymph nodes: malignant or not? *Endoscopy* **24**: 320–323

Grimm H, Binmoeller KF, Hamper K, Koch J, Henne Bruns D and Soehendra N (1993) Endosonography for preoperative locoregional staging of esophageal and gastric cancer. *Endoscopy* **25**: 224–230

Guhlmann A, Storck M, Kotzerke J, Moog F, Sunder Plassmann L and Reske SN (1997) Lymph node staging in non-small cell lung cancer: evaluation by [$^{18}$F]FDG positron emission tomography (PET). *Thorax* **52**: 438–441

Hamacher K, Coenen HH and Stocklin G (1986) Efficient stereospecific synthesis of no-carrier-added 18F-fluoro-2-deoxy-D-glucose using aminopolyether supported nucleophilic substitution. *J Nucl Med* **27**: 235–238

Kemeny MM, Hogan JM, Ganteaume L, Goldberg DA, Terz JJ (1986) Preoperative staging with computerized axial tomography and biochemical laboratory tests in patients with hepatic metastases. *Ann Surg* **203**: 169–172

Lehr L, Rupp N and Siewert JR (1988) Assessment of resectability of esophageal cancer by computed tomography and magnetic resonance imaging. *Surgery* **103**: 344–350

Lightdale CJ (1992) Endoscopic ultrasonography in the diagnosis, staging and follow-up of esophageal and gastric cancer. *Endoscopy* **24**: 297–303

Masurin VS, Ryndin VD, Efimov ON and Allahverdjian AS (1992) Surgery of localized cardioesophageal cancer. *Semin Surg Oncol* **8**: 33–36

Molloy RG, McCourtney JS and Anderson JR (1995) Laparoscopy in the management of patients with cancer of the gastric cardia and oesophagus. *Br J Surg* **82**: 352–354

Moreaux J and Horiot A (1980) [Adenocarcinoma of the cardia and proximal third of the stomach. Results of surgical treatment in 80 consecutive cases]. *Gastroenterol Clin Biol* **4**: 758–764

Plukker JT, Mulder NH, Sleijfer DT, Grond J and Verschueren RC (1991) Chemotherapy and surgery for locally advanced cancer of the cardia and fundus: phase II study with methotrexate and 5-fluorouracil. *Br J Surg* **78**: 955–958

Plukker JT, Sleijfer DT, Verschueren RC, Van der Graaf WT and Mulder NH (1995) Neo-adjuvant chemotherapy with carboplatin, 4-epiadriamycin and teniposide (CET) in locally advanced cancer of the cardia and the lower oesophagus: a phase II study. *Anticancer Res* **15**: 2357–2361

Rahamim J and Cham CW (1993) Oesophagogastrectomy for carcinoma of the oesophagus and cardia. *Br J Surg* **80**: 1305–1309

Saunders HS, Wolfman NT and Ott DJ (1997) Esophageal cancer. Radiologic staging. *Radiol Clin North Am* **35**: 281–294

Siewert JR and Dittler HJ (1993) Esophageal carcinoma: impact of staging on treatment. *Endoscopy* **25**: 28–32

Stahl M, Wilke H, Fink U, Stuschke M, Walz M, Siewert R, Molls M, Fett W, Makoski HB, Breuer N, Schmidt U, Niebel W, Sack H, Eigler FW and Seeber S (1996) Combined preoperative chemotherapy and radiotherapy in patients

 © Cancer Research Campaign 1998

with locally advanced esophageal cancer: interim analysis of a phase II trial. *J Clin Oncol* **14**: 829–837

Steinert HC, Hauser M, Allemann F, Engel H, Berthold T, von Schulthess GK and Weder W (1997) Non-small cell lung cancer: nodal staging with FDG PET versus CT with correlative lymph node mapping and sampling. *Radiology* **202**: 441–446

Strauss LG (1996) Fluorine-18 deoxyglucose and false-positive results: a major problem in the diagnostics of oncological patients. *Eur J Nucl Med* **23**: 1409–1415

Sussman SK, Halvorsen RA, Illescas FF, Cohan RH, Saeed M, Silverman PM, Thompson WM and Meyers WC (1988) Gastric adenocarcinoma: CT versus surgical staging. *Radiology* **167**: 335–340

Tio TL, Cohen P, Udding J, den Hartog-Jager FC and Tytgat GNJ (1989) Endosonography and computed tomography of esophageal carcinoma. *Gastroenterology* **96**: 1478–1486

Valk PE, Pounds TR, Hopkins DM, Haseman MK, Hofer GA, Greiss HB, Myers RW and Lutrin CL (1995) Staging non-small cell lung cancer by whole-body positron emission tomographic imaging. *Ann Thorac Surg* **60**: 1573–1581

Vilgrain V, Mompoint D, Palazzo L, Menu Y, Gayet B, Ollier P, Nahum H and Fekete F (1990) Staging of esophageal carcinoma: comparison of results with endoscopic sonography and CT. *AJR Am J Roentgenol* **155**: 277–281

Vitola JV, Delbeke D, Sandler MP, Campbell MG, Powers TA, Wright JK, Chapman WC and Pinson CW (1996) Positron emission tomography to stage suspected metastatic colorectal carcinoma to the liver. *Am J Surg* **171**: 21–26

Watt I, Stewart I, Anderson D, Bell G and Anderson JR (1989) Laparoscopy, ultrasound and computed tomography in cancer of the oesophagus and gastric cardia: a prospective comparison for detecting intra-abdominal metastases. *Br J Surg* **76**: 1036–1039

Yasuda S, Raja S and Hubner KF (1995) Application of whole-body positron emission tomography in the imaging of esophageal cancer. *Surg Today* **25**: 261–264

Yoshioka T, Takahashi T, Oikawa H and Kanamaru R (1994) [Experimental study on the effectiveness of PET tumor images for cancer diagnosis]. *Gan To Kagaku Ryoho* **21**: 369–373

Ziegler K, Sanft C, Zeitz M, Friedrich M, Stein H, Haring R and Riecken EO (1991) Evaluation of endosonography in TN staging of oesophageal cancer. *Gut* **32**: 16–20

© Cancer Research Campaign 1998